# Vital Force

**Published:** 1884-01

**Authors:** P. P. Wells

**Affiliations:** Brooklyn


### VITAL FORCE.

P. P. Wells, M. D., Brooklyn.

[*Read before Kings County Homœopathic Medical Society, Dec. 4th, 1883.*]

"Is there any such thing as the vital force? * * * * It is quite universally decided by modern scientists that there is no such thing as vital force."—*Medical Advance*, Nov., 1883.

It is not a very exalted employment, that of attempting to demonstrate self-evident facts! If the question were asked, Is light from the sun? it would be a sufficient answer to reply—Look and see. That light is from the sun is not a more self-demonstrated fact than is this other, the existence of which we are told is "universally denied by modern scientists." It is demonstrated in the execution of every function of each organ of the body. In sanguification, circulation, digestion, absorption, assimilation, nutrition, excretion, secretion, voluntary and involuntary motion, sensation, thought, emotion, as well as in whatever of phenomena are manifested in the organism which distinguish the living from the dead man. And yet we are told "modern scientists universally deny" the existence of this self-demonstrated fact! What then? That which is most evident, as the outcome of this silly denial is, that it is so much the worse for these negators, who style themselves *scientists*. No *fact* can be extinguished by negation, and, least of all, facts which demonstrate themselves in every phenomena of *living* existences. Such facts remain, deny them who will, the same after as before negation. And this fact, said to be "universally denied," by men who should know better, will continue as long as distinctions exist between the living and the dead, and long after these scientific negators have ceased to exhibit, in their own persons, this characteristic of universal life. It depends for its existence on no man's recognition or acceptance. It cannot be blotted out by negation, though universal, even by self-styled "scientists."

What is that in the human organism which, while present in it, preserves its parts in integrity of tissue and function, which, when removed, these pass under the dominion of laws that reduce the whole to destructive dissolution? While present, this something, whatever it is, protects the material tissues from the action of chemical laws which control all other forms of matter, *i. e.*, all matter not pervaded by life; but this something removed, the material body passes under these laws, and by them into its constituent elements, and organism is no more to be predicated of them than if they had never entered into the constitution of a moving, thinking, sentient organization. What is this, the presence or absence of which is attended by such vast consequences? It is that, and that only, which we are told "modern scientists universally deny" having any existence at all. Let them, then, account for this preservation and this dissolution, excluding from the problem this rejected fact, if they can. The man who but a moment since was before us in perfect exhibition of bodily and mental functions, now is seen, by God's providence, or accident, if the expression be preferred, deprived instantly of all these, and yet the material body is as perfect in all its parts as before. He is, as to all perceptible physical facts, the same he was when these functions were active, and yet these have all stopped. What has happened?

Matter pervaded by life presupposes matter in motion. In the living human body, the aggregate of these motions are summed up in the word function. All the functions of bodily organs are the results of the motions characteristic of life. Now, motion implies a motive power. To predicate motion without such power or force is to perpetrate an absurdity in philosophy than which no greater can be imagined. It is not forgotten that one at least of these "scientists"* has attempted to explain these functional movements by referring them to the action of cell walls, intercellular contents and spaces on each other. That this attempted explanation is wholly inadequate is apparent from the fact that in the dead man we have supposed, cell walls, contents, and interspaces are the same, so far as human perception can see, after functions have ceased as before, and yet all motion has stopped, which, if the motive power were inherent in these physical parts, and relations, and functions only resulting from the action of these on each other, as this attempted explanation assumes, it should not have done. On the contrary, while these parts and relations continue, motion (function) should go on. In other words, cell walls, intercellular spaces and contents continuing, life must continue with all its functional motions, and death be a physical impossibility. It will be remembered, these functions have stopped, and men have died all the same, since this fanciful explanation, as before, which would have been impossible if the motive power of function had been inherent in these physical parts and relations. The parts and relations continuing intact, motion derived from their action and reaction on each other must be perpetual, in the very nature of things. But motion and function stop, and the explanation stands before the fact convicted of inadequacy and stupidity. At the same time another power is demonstrated, which pervades these cells, etc., wholly distinct from them, and by which these are moved and controlled. This other power it is to which we refer when we speak of *vital force.* There need be no hesitation in affirming its existence, or in ascribing to it the execution of all the functions of the living body. There need be no hesitation in recognizing this power as inseparable from all life, and which the negations of all so-called "scientists" are as incapable of removing from the phenomena of life, as they are of removing the Alps to the midst of the sea.

This power it is, then, which governs and executes all the functions of life. In this view, then, its importance becomes appa-

---

* Von Grauvogle.

rent. It is seen at once that a just view of this power must underlie all *true pathology*. This being the science of *sick* life, and the functions of life being wholly under the control of this power, it follows that sickness is primarily a disturbance of this power as revealed in the changed functions, now only imperfectly performed, or not performed at all, because if the impress of the morbid cause on this power, by which its action on organs and functions is changed from that balance of the action of each on the other which is conservative of the whole, and of all parts of the body, which we call health, to that loss of this balance, which we call disease. This is just what sickness is, and denying the existence of this power and excluding it from all views of sicknesses, it is no wonder that those who affect a pathology from which this power is excluded should find this science, so eviscerated, practically "*misleading*."* And then it should be borne in mind that those who refuse to recognize this power in their physiology and pathology have brought to our knowledge no substitute by which they would have us understand how the processes of life are executed. The whole is left blank by them as to any executive power by which life functions are effected, either in health or sickness. They leave the whole in dead negation and confusion. While denying this explanation of health and sickness, given by Hahnemann, they give us no other or better in its place. They, at least, some of them, affect a pathology which regárds diseases as a kind of entities, things distinct from the suffering individual, to be marked out and *named*, and the first great duty of the prescriber is done, though this thing, which they call pathology, has its only existence in their own unenlightened imagination.

It is not difficult to see then how a knowledge of this power underlies all true philosophy of health and sickness, as, most certainly it does, the philosophy of Homœopathy. It is the one great starting-point from which all rational investigation of both must begin. To deny its existence is to cut off the only light in which each can be distinctly seen and understood. "*Scientists*" may do this, if they will, but in so doing they displace the chief corner-stone of the only true sciences of pathology and therapeutics, which certainly is not a work a true "*scientist*" should be proud of.

But says the *Advance*, "The facts of Homœopathy are one thing, the philosophy of Homœopathy is quite another." Just so. Just as the foundation and superstructure of a building are

* W. S. Searle, in *North American Journal of Homœopathy*.

not identities, but they are inseparable, and the one without the other is void of all utility. The *philosophy* of Homœopathy rests on its *facts*, and this it is which renders its standing impregnable. And it is this chief corner-stone of its foundation, a self-demonstrating and self-demonstrated fact, which has so largely sustained its philosophy and its practice in the hands of those whose successes have given this practice a world-wide repute and acceptance. It should not be forgotten, that it is not those who have been foremost and loudest in proclaiming that Hahnemann was guilty of fallacies, errors, and ignorance, and who have placed this fundamental power in healthy and sick life as one of his fallacies, who have contributed aught to the successes which have carried the philosophy and practice of this great man over the civilized world.

We have seen the importance of the fact we are discussing, the *Vital Force*, to all right understanding of life, sick and in health. We have found it the chief corner-stone of homœopathic philosophy, and we may add, without this force as a chief factor, Homœopathy *has no philosophy*,* neither can there be, without this force included in it, any rational philosophy of life, health, or sickness. We shall now go farther, and show, if we may, that a right recognition and knowledge of this force is no less indispensable to a useful and successful practice of this philosophy.

The practice of the homœopathic philosophy is the curing of sicknesses by applying agents to this end, in accordance with the law of similars. The first duty then, in this practice, is to obtain a clear view of the facts (totality of the symptoms) of the sickness which we are to cure. They are to be found in the changed function or functions of some organ or organs, from that harmonious action which we call health, to dissonance of this harmony which we call sickness. Now, this force which we have been discussing, we have seen to be the governor and executant of all functional action, and, therefore, if this harmony be changed, the impress of the changing cause must have been primarily on this governing and executing force, and the changed

---

* It has been said of one who was combating an argument of one of our ablest homœopathists, that he thought when he had refuted this principal fact in his opponent's argument, the vital force, that Homœopathy itself would "topple to the ground," and he was quite right. The philosophy, and all connnected with it, goes when that is taken away on which it principally rests. The great difficulty is, in this argument, to displace a self-evident fact, which the Almighty has planted in the very foundation of His philosophy of life and therapeutics. Negation, even by so-called "scientists," is not adequate to the task.

functions are the result. It is not then a *material thing*, distinct from the suffering body, with which we have to do, but a condition resulting from change of action of this force, which has wrought change of function, *i. e.*, sickness. This factor in the problem of healing, the group of morbid phenomena, or the totality of the symptoms, then, is an immaterial factor, a changed mode of action.

The second duty is, to find in the record of the materia medica that agent which has been found by actual experiment to have produced similar changes of function in those who were in health when the drug was taken. That one which has produced changes the *most* similar is the most certain cure. And we may add, and we do so with confidence, the result of long practical experience of the truth of this fact, that the greater the similarity of these two factors in our problem of curing, the higher should be the potence employed, and the cure will be the more certain, rapid, and perfect. The immaterial nature of the morbific cause, acting on this immaterial force, which controls function, producing changes in this which are also immaterial. These changes are best met (the most like having been ascertained) by potencies of the agent which are also immaterial, which those we now call "*high*" certainly are.

Here, then, is the view of pathology which belongs to Homœopathy, as a part of its distinctive philosophy. It was first promulgated by Hahnemann, who has, from the beginning of his career till now, been libellously accused of having "*no pathology*," and this though he proclaimed the only rational pathology the world has known, at the same time he gave his science of therapeutics, which is in so beautiful harmony with this pathology. This pathology should stand in sharp contrast with that *pseudo* pathology which from a few generic facts predicates an internal condition, gives this a *name*, and so erects an objective for practical effort. It imagines a condition, *i. e.*, guesses at it—and then imagines drug A, B, or C will remove this condition, *i. e.*, guesses at it—gives the drug, which very likely produces more of sickness than it cures, and then those who thus practice cry out, Behold "*scientific medicine!*" It will be seen here that each element in the prescription is a *guess*, and nothing better. As opposed to this, each element in a *true* homœopathic prescription is a fact which may be *positively known*. What, in view of the above discussion, can the "*scientist*" have known or been thinking of when he denied the existence of a vital force? Is the denial calculated to increase our respect for so-called "*scientists*"?

But it is said—

> "He who says Homœopathy as a science is the law of vital force, is talking in a language common enough half a century ago, but wholly out of date to-day."

How out of date? The "vital force" is a fact, or life is not a fact. To talk of life without force is to talk of a life which is only a synonym of death. We have seen the life-force to be a *fact*, and we have seen it in the execution of its offices. Then how is speaking of it out of date? Do *facts* become "*old fashioned*"? The fact is certainly an old one, dating its existence back to the creation of the first man of the race, and will continue its offices till life in the last has ceased. It has never, that we know of, been superseded in its offices by any other fact or force; and to talk of this now is no more out of date than it was "half a century," or half a hundred centuries, ago. Facts in nature do not often become obsolete. This one never has, but is still active and beneficent as it was when first planted in the being of the progenitor of our race. Did the writer mistake "out of date" for "out of fashion," accepting these "modern scientists" (who often *know* less than they think they do) as the arbiters of fashion? Truth may become unfashionable, but never "out of date." It may become unfashionable, but then it is only so much the worse for the fashion. That so important a truth as this should become so, a truth which makes so important an integer in all rational philosophy of life, sick or in health, *i. e.*, in all rational physiology and pathology, and a right understanding of which enters so largely into all intelligent treatment of sicknesses, is a reproach to the present current of thought and to the teachings and influences from which this fashion of discarding truth has arisen. It is to be remembered with gladness that we have still with us some who retain a knowledge of this truth and the courage to assert it, though the fashion of so-called "scientists" may be disposed to thrust it aside with scorn.

The *Advance* complains of the definition of disease as the "impairment of the equalization of the vital force." If it has a better or more intelligible definition of the facts of disease, let it be given, and let it have all the consideration it merits. In the absence of all other definitions we accept this. It comprehends in itself the facts of the matter defined, and we see no wisdom in abandoning this till a better is offered. In the meantime, till a better is given, will it not be well to stop the cry, of which we think we have now had more than enough, of "no pathology"?

